# Mesenchymal Stem Cell‐Derived Exosomes Improve Aging‐Related Changes in Liver Lipid Metabolism by Enhancing Autophagy

**DOI:** 10.1111/acel.70642

**Published:** 2026-07-16

**Authors:** Jinquan Li, Mengqi Gao, Jinke Feng, Dini Huo, Rui Hong, Fuhua Zhang, Qin He, Ming Dong

**Affiliations:** ^1^ Department of Endocrinology and Metabolism Qilu Hospital of Shandong University Jinan China; ^2^ Shandong Provincial Key Laboratory of Spatiotemporal Regulation and Precision Intervention in Endocrine and Metabolic Diseases Jinan China; ^3^ Shandong Provincial Engineering Research Center for Advanced Technologies in Prevention and Treatment of Chronic Metabolic Diseases Jinan China; ^4^ Institute of Endocrine and Metabolic Diseases of Shandong University Jinan China

**Keywords:** autophagy, exosomes, lipid metabolism, liver, senescence

## Abstract

Health problems associated with aging have become increasingly severe in recent years. For example, hepatic lipid metabolism declines as the body ages, leading to lipid metabolic disorders. Although exosomes have been explored for treating metabolic diseases, there is currently a paucity of research regarding aging‐related changes in hepatic lipid metabolism. Herein, we cultured human umbilical cord mesenchymal stem cells (HucMSCs), from which we extracted HucMSC‐derived exosomes (HucMDEs). We then established a natural aging mouse model in vivo and a palmitic acid‐induced AML12 cell model in vitro; HucMDEs were subsequently used as an intervention. Western blot analysis and quantitative real‐time PCR were used to investigate changes in liver lipid metabolism and senescence‐related markers in vivo and in vitro. Our results revealed that the HucMDE‐injected mice showed reduced body weights, increased insulin sensitivity, decreased hepatic lipid deposition, reduced senescence, and augmented autophagy levels compared with the aged group of mice. The in vitro results were consistent with the in vivo results. When autophagy‐related genes were silenced in AML12 cells via small interfering RNAs, or when cells were treated with the autophagy inhibitor 3‐methyladenine or the lysosomal inhibitor bafilomycin A1, HucMDEs were able to reverse the reduction in hepatocyte autophagy levels. In this study, we demonstrated that HucMDEs improved hepatic lipid metabolism and attenuated cellular senescence by enhancing autophagy. We expect that these findings will provide novel potential therapeutic targets for the treatment of hepatic lipid metabolism disorders during aging.

## Graphical abstract

Schematic summary of the protective effects of HucMSC‐derived exosomes (HucMDEs) against aging‐related hepatic lipid metabolism disorders through autophagy enhancement.

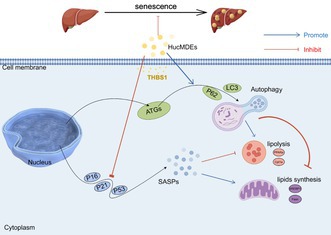

## 1 Introduction

Cellular senescence is a multifaceted process involved in both the physiological aging process and the pathogenesis of various chronic human diseases. This process has attracted increasing scientific attention in recent years, with emerging evidence suggesting that senescence plays a critical role in the initiation and progression of hepatic lipid accumulation. Notably, the transition from nonalcoholic fatty liver disease (NAFLD) to nonalcoholic steatohepatitis (NASH) is frequently accompanied by key pathophysiological alterations, including metabolic dysfunction and inflammatory responses. These pathophysiological changes are both driven by cellular senescence and in turn exacerbate it, making senescence both a precipitating factor and an exacerbating mediator of NAFLD progression (Baboota et al. 2022; Engelmann and Tacke 2022; He and Sharpless 2017; Nassir 2022).

Although the primary therapeutic strategies for hepatic lipid metabolism disorders associated with aging state are currently dietary control and lifestyle improvement, several pressing challenges remain unaddressed, including the lack of specific therapeutic agents, the limited variety of drugs available for clinical trials, and the lack of approved drugs for NASH and liver fibrosis (Nassir 2022). Therefore, investigations that explore effective treatments for hepatic lipid metabolism disorders in the aging state are warranted.

With the development of regenerative medicine centered on stem cell technology, mesenchymal stem cells (MSCs) are being widely used to treat a variety of diseases (Uccelli et al. 2008), and MSC‐derived exosomes represent a key paracrine effector of MSCs. MSC‐derived exosomes can exert therapeutic effects while avoiding the risks of stem cell transplantation and eliciting a weaker host immune response (Abbaszadeh et al. 2020; Keshtkar et al. 2018; Tang et al. 2021). Recent research has suggested that MSC‐derived exosomes improve hepatocellular function (Lou et al. 2017) and attenuate inflammatory responses by reducing immune cell infiltration and pro‐inflammatory cytokine production, both of which are topics of intense current interest. However, relatively little is known regarding the role of MSC‐derived exosomes and their underlying mechanisms of action in aging‐related liver injury.

Autophagy is a crucial intracellular degradative process that enables cells to maintain cellular homeostasis. Dysfunctional autophagy in liver cells can lead to a variety of liver diseases, including NAFLD, alcohol‐related liver disease, cirrhosis, and liver cancer (Qian et al. 2021). Accumulating evidence indicates that autophagy mediates the degradation of hepatic lipid droplets (Sakane et al. 2021; Singh et al. 2009). Autophagy is hypothesized to play a key role in the development of hepatic lipid metabolism disorders, and some studies have suggested that the inhibition of autophagy is a significant contributing factor to these disorders (González‐Rodríguez et al. 2014; Yan et al. 2022). Exosomes have been shown to improve liver fibrosis and mitigate drug‐induced liver injury by enhancing autophagy; however, whether exosomes ameliorate aging‐associated liver injury via the autophagy pathway remains unclear (Gao, Wei, et al. 2020; Li, Gong, et al. 2023).

Herein, we established a natural aging C57BL/6 mouse model in vivo and a palmitic acid‐induced cellular model in vitro to investigate the role and underlying mechanisms of human umbilical cord mesenchymal stem cell‐derived exosomes (HucMDEs) in aging‐related hepatic lipid metabolism. Our findings aim to provide a theoretical basis and therapeutic strategy for the management of aging‐associated hepatic lipid metabolic disorders.

## 2 Methods

### 2.1 Extraction and Identification of HucMSCs

The study was approved by the Ethics Committee of Qilu Hospital of Shandong University (DWLL‐2022‐090). Umbilical cord samples for the isolation of human umbilical cord mesenchymal stem cells (HucMSCs) were collected from five full‐term infants delivered via cesarean section by young, healthy mothers at the Department of Obstetrics and Gynecology. Fresh umbilical cord samples were stored in sterile containers containing culture medium (MEMɑ) and washed with sterile phosphate‐buffered saline (PBS) supplemented with penicillin and streptomycin to remove residual blood. The outer amniotic membrane was bluntly dissected to expose Wharton's jelly, and umbilical arteries and veins were removed. The Wharton's jelly was minced into small pieces, the finely chopped tissue mass was evenly placed on the bottom of the culture flask, and complete medium was slowly added to the bottle to continue the culture, with normal medium refreshed every 2–3 days.

HucMSCs cultured to passage 3–4 were digested with trypsin, centrifuged at 178 g for 5 min at 4°C, washed with PBS, resuspended in 500 μL of PBS, and evenly dispensed into flow tubes. Cells in four separate tubes were incubated with 2 μL of each single‐labeled antibody (CD34, CD73, CD105, and HLA‐DR) for 30 min at room temperature in the dark, with gentle mixing every 10 min, then centrifuged for 2–4 min at 178–715 g. An unstained cell suspension served as the blank control. PBS was then added to wash the cells once, and the cells were ultimately resuspended in each tube with 500 μL of PBS and evaluated by flow cytometry (BD, Accuri, Bergen, NJ, USA). Detailed antibody information is provided in Data S1.

HucMSCs are capable of multilineage cell differentiation. Osteogenic differentiation was induced using medium supplemented with fetal bovine serum, penicillin–streptomycin, glutamine, ascorbic acid, sodium β‐glycerophosphate, and dexamethasone. HucMSCs at 80%–90% confluence were digested with 0.25% trypsin. The digested cells were seeded into six‐well plates and cultured at 37°C in a 5% CO_2_ humidified cell culture incubator. When 60%–70% of the cells in the six‐well plate were confluent, the complete medium in the wells was aspirated, induction medium was added, and the medium was changed every 3 days. After 2–4 weeks of induction, the morphology and growth of the cells were observed, the induction of differentiation was halted, and Alizarin Red staining was performed.

For adipogenic differentiation, HucMSCs at 80%–90% confluence were digested with 0.25% trypsin and seeded into six‐well plates. Adipogenic induction medium was prepared with fetal bovine serum, penicillin–streptomycin, glutamine, insulin, rosiglitazone, IBMX, and dexamethasone. Cells were cultured in 2 mL of complete medium per well at 37°C in a 5% CO_2_ humidified incubator. When cells reached 90%–100% confluence, the medium was replaced with induction medium, which was refreshed every 2–3 days. Induction was maintained for 12–18 days before termination. When the formation of lipid droplets in the cells could be observed under the microscope (OLYMPUS, IX53, Tokyo, Japan), the induction of differentiation was stopped, and Oil Red O staining was subsequently performed.

### 2.2 Isolation and Identification of Exosomes and Tracking of Exosomes

When 70%–80% of the human embryonic lung fibroblasts (HELFs) and HucMSCs were confluent, the culture medium was changed to serum‐free medium. After 48 h of incubation, the conditioned medium was collected and centrifuged to remove cells and cellular debris, and the supernatant was subsequently filtered through a 0.22 μm filter. Finally, the supernatant was ultracentrifuged at 120,000 *g* for 75 min at 4°C to obtain exosomes (Beckman, XPN‐100, Brea, CA, USA). The newly extracted exosomes were then fixed by the addition of glutaraldehyde solution and immediately sent to the transmission electron microscopy (TEM; FEI, Tecnai G2 Spirit BioTwin, Hillsboro, OR, USA) center for preparation and imaging. We conducted nanoparticle size analysis with a nanoparticle tracking analyzer (NTA; Particle Metrix, ZetaView, Meerbusch, Germany), and the exosome markers HSP70 and TSG101 were detected by western blot (WB) analysis. The primary antibodies we used are shown in Data S1. The protein concentration of the isolated exosomes was determined using a BCA protein assay kit (Beyotime, China).

To trace the exosomes in vivo, we labeled them with Cy7 and injected them into mice via the tail vein. Afterward, the exosomes were tracked using an in vivo imaging system (IVIS; Tanon, ML500, Shanghai, China). To trace the exosomes in vitro, the exosomes were labeled with PKH67 (green fluorescence; Merck, PKH67GL, Darmstadt, Germany). Subsequently, the labeled exosomes were diluted with PBS and co‐incubated with AML12 cells for 24 h. Cells were then washed twice with PBS for 3 min each. The cells were fixed with immunostaining fixative for 30 min at room temperature, and the nuclei of the cells were stained with DAPI. Cytoskeletons were visualized using rhodamine phalloidin (Cytoskeleton, Thermo Fisher, no. R415, Waltham, MA, USA). Fluorescent images were captured using a fluorescence microscope (OLYMPUS, BX53, Tokyo, Japan).

### 2.3 Animals

Eight‐week‐old male C57BL/6 mice were housed under standard conditions with free access to standard chow (Xietong Pharmaceutical Bio, no. 1010112, Nanjing, China) and water. Bedding was replaced and cages were cleaned at regular intervals. Subsequently, the mice were randomly divided into three groups: young control group (Young, 8 weeks old), aged group (Old, 18 months old), and aged group treated with HucMDEs (Old+HucMDEs). Exosomes from HucMSCs were injected into the mice via the tail vein at a dose of 5 μg/g every 3 days for 12 weeks, and the Old group was simultaneously injected with an equal volume of PBS via the tail vein (Figure 2A). Body weight (BW) was monitored throughout. The levels of alanine aminotransferase (ALT), aspartate aminotransferase (AST), triglyceride (TG), and total cholesterol (TC) in mouse serum were determined according to the instructions of the ELISA kits (nos. C009‐2‐1, C010‐2‐1, A110‐1‐1, and A111‐1‐1, respectively; NJJCBIO, Nanjing, China).

### 2.4 Intraperitoneal Glucose Tolerance Test (IPGTT) and Intraperitoneal Insulin Tolerance Test (IPITT) Procedures

Measurements were performed 1 week after the last exosome and PBS injection, and IPGTT and IPITT were performed at 2‐ to 3‐day intervals. During IPGTT and IPITT, the mice fasted for 14–16 h and 4–6 h, respectively. Mice had free access to water during fasting. A 50% glucose solution (Solarbio, no. G8150, Beijing, China) and insulin solution (Beyotime, no. P3376, Shanghai, China) were prepared in advance and filtered through a sterile filter on an ultraclean bench. Fasting blood glucose levels and body weights were measured at baseline. Mice then received an intraperitoneal injection of glucose at 2 mg/g body weight for IPGTT or insulin at 0.75 IU/kg for IPITT. The blood glucose levels of the mice were determined at 30, 60, 90, 120, and 180 min, and the data were recorded and then statistically analyzed.

### 2.5 Cell Culture and Treatments

Human embryonic lung fibroblast (HELF) was purchased from the China Cell Culture Center (Shanghai, China) and cultured in DMEM (Hyclone, UT, USA) supplemented with 10% fetal bovine serum (FBS) and maintained at 37°C under 5% CO_2_. For HucMSCs culture, the medium was changed every 2–3 days according to the density of cell growth, passaging, and passaging ratio (the latter primarily at 1:3). Culture conditions were 37°C and 5% CO_2_ in humidified compressed air. The basal culture medium consisted of MEMα (Thermo Fisher, no. 12571063, Waltham, MA, USA) supplemented with FBS, penicillin–streptomycin, epidermal growth factor (EGF; Novoprotein, no. C029, Suzhou, China), and fibroblast growth factor (FGF; Novoprotein, no. C779, Suzhou, China). The HELFs culture conditions were 37°C and 5% CO_2_ as above, using DMEM (Thermo Fisher, no. 11965092, Waltham, MA, USA) supplemented with FBS and penicillin–streptomycin. The Alpha Mouse Liver 12 (AML12) normal mouse hepatocyte cell line (Procell, no. CL‐0602, Wuhan, China) was cultured under the same temperature and CO_2_ conditions. The basal medium was DMEM/F12 supplemented with FBS, penicillin–streptomycin, insulin, transferrin, selenium, and dexamethasone (AML12 cell‐specific culture medium; Procell, no. CM‐0602, Wuhan, China). A cellular model of lipid deposition and senescence was established by treating AML12 cells with palmitic acid (PA; 0.5 μM, Sigma‐Aldrich, no. P9767, Darmstadt, Germany), as previously described (Chen et al. 2024; Lee et al. 2021; Zhang et al. 2022). AML12 cells were seeded into six‐well plates and divided into different treatment groups: control, PA, PA + HELF‐derived exosomes (HEDEs), PA + HucMDEs, PA + 3‐methyladenine (3‐MA, 10 mM, Sigma‐Aldrich, no. 189490, Darmstadt, Germany), and PA + bafilomycin A1 (Baf, 30 nM, no. B1793, Sigma‐Aldrich, Darmstadt, Germany). All treatments lasted for 24 h. AML12 cells with small interfering RNA (siRNA), the siRNA sequences used are shown in Data S1. AML12 cells were transfected with autophagy‐related genes using Lipofectamine 2000 transfection reagent according to the manufacturer's instructions (Thermo Fisher, no. 11668027, Waltham, MA, USA). The medium was removed and replaced with Opti‐MEM I reduced serum medium (Thermo Fisher, no. 31985070, Waltham, MA, USA) and mixed with siRNA for autophagy‐related genes (ATGs) for 6 h. Subsequently, the reduced serum medium containing Opti‐MEM I, mixed with siRNA targeting ATGs, was replaced with complete culture medium, and AML12 cells were treated with HucMDEs. After 24 h, the AML12 cells were collected for subsequent experiments.

### 2.6 Autophagic Flux Assay

AML12 cells were transfected with RFP‐GFP‐LC3 (Beyotime Biotechnology, Shanghai, China) for 24 h to detect autophagy flux, as previously described (Zeng et al. 2022). Red fluorescent LC3 protein indicated that the autophagosomes were engulfed in the lysosomes to form an autolysosome. Green fluorescent LC3 protein indicated that the autophagosomes could not be converted to autolysosomes, suggesting that the autophagy process was blocked.

### 2.7 Alizarin Red Staining

After removing the induction medium, the HucMSCs were washed with PBS 1–2 times, then fixed with neutral formaldehyde. After the removal of neutral formaldehyde and rinsing with PBS, 2 mL of Alizarin Red staining solution (Solarbio, no. G1452, Beijing, China) was added to each well for 30 min. After removing the staining solution and rinsing each well with PBS, HucMSCs were observed using a light microscope (OLYMPUS, IX53, Tokyo, Japan) and photographed.

### 2.8 Hematoxylin–Eosin (H&E) Staining

Paraffin sections of the mouse livers were heated in an oven and dewaxed with xylene and an ethanol gradient. After the sections were washed with distilled water, they were stained with hematoxylin dye (Servicebio, no. G1004, Wuhan, China) and eosin dye solution (Servicebio, no. G1002, Wuhan, China). After the dye was removed, the sections were dehydrated, sealed, observed, and photographed under a microscope (OLYMPUS, BX53, Tokyo, Japan).

### 2.9 Senescence‐Associated‐β‐Galactosidase (SA‐β‐Gal), Periodic Acid‐Schiff (PAS), Oil Red O Staining

According to the kit instructions (Beyotime, no. C0602, Shanghai, China) for SA‐β‐Gal staining, the dyeing solution was added to the frozen liver tissue sections and cultured cells, and the samples were incubated at 37°C overnight. PAS staining of the frozen liver tissue sections was performed per the instructions of the PAS staining kit (Servicebio, no. G1281, Wuhan, China). Cultured cells were stained per the specialized kit instructions (Servicebio, no. G1360, Wuhan, China). Oil red O staining of frozen mouse liver slides was performed using Oil Red O kits (Solarbio, no. G1261, Beijing, China and Solarbio, no. G1262, Beijing, China) specialized for cultured cells. The images were then observed under a microscope.

### 2.10 Protein Extraction and WB Assay

Total protein was extracted from mouse liver tissues and cultured cells. Tissues were homogenized at low temperature, and cell cells were lysed by sonication (SCIENTZ, No. JY92‐IIN, Ningbo, China). Samples were then centrifuged (Eppendorf, no. 5424R, Hamburg, Germany), and the supernatant was collected. The protein samples were separated using SDS–PAGE (Epizyme, no. PG112&PG113, Shanghai, China). Proteins were then transferred to PVDF membranes (Millipore, No. ISEQ00010, Billerica, Massachusetts, USA) and incubated with an antibody against the target protein overnight at 4°C. The primary antibodies are shown in Data S1.

### 2.11 Immunohistochemistry (IHC)

Paraffin‐embedded mouse liver sections were baked at 65°C for 1 h. After retrieving the antigen, the slides were incubated with the corresponding primary antibody at 4°C overnight. The next day, sections were incubated with a secondary antibody, and DAB reagent (Genetech, No. GK600511, China) was used for immunohistochemical staining.

### 2.12 RNA Extraction and Quantitative Real‐Time PCR (qPCR)

Total cellular RNA was extracted using an RNA extraction kit (AGbio, no. 21011, Changsha, China) according to the manufacturer's instructions. After gDNA removal, the RNA was transferred to a thermal cycling apparatus for reverse transcription (Applied Biosystems, no. 2720, Waltham, MA, USA). The qPCR system (Roche, Light Cycler 480, Swiss) was prepared according to the kit instructions (AGbio, No. 11701, Changsha, China), and the qPCR conditions were set to perform the reactions. The primer sequences for the genes are shown in Data S1.

### 2.13 Proteomics

Proteomics was performed at Tsingke Biotech Co. Ltd (Beijing, China). DIA‐NN was employed for the qualitative and quantitative analysis of raw DIA data. Statistical analyses were performed to identify biologically meaningful variations: SciPy was utilized to assess the significance of differential expression and conduct correlation analysis (to investigate associations between samples or features). Additionally, statsmodels was applied for multiple hypothesis testing to control the false discovery rate (FDR), thereby improving the reliability of statistical inferences.

### 2.14 RNA‐Seq

Total RNA was extracted from AML12 treated with PA and PA+HucMDEs using Trizol reagent (Invitrogen, USA) according to the manufacturer's instructions. Library construction and sequencing were performed at Tsingke Biotech Co. Ltd. The sequence alignment of clean reads to the reference genome was performed using Hisat2. Significant differences between samples were analyzed using DEseq2 for downstream pathway analysis.

### 2.15 Statistical Analysis

The independent experiments were repeated at least three times. Statistical analysis was performed using GraphPad Prism 9 (San Diego, CA, USA). Data are expressed as the mean ± standard error of the mean (SEM). Differences between the two groups were compared using an unpaired Student's *t*‐test. Differences among the three groups were analyzed using one‐way ANOVA followed by Tukey's post hoc test for multiple comparisons. A *p* value < 0.05 was considered to indicate statistical significance, ns indicates *p* > 0.05, and significance was expressed as follows: *p < 0.05, **p < 0.01, and ***p < 0.001.

## 3 Results

### 3.1 Identification of HucMSCs and HucMDEs, and Tracking of HucMDEs In Vivo and In Vitro

**FIGURE 1:**
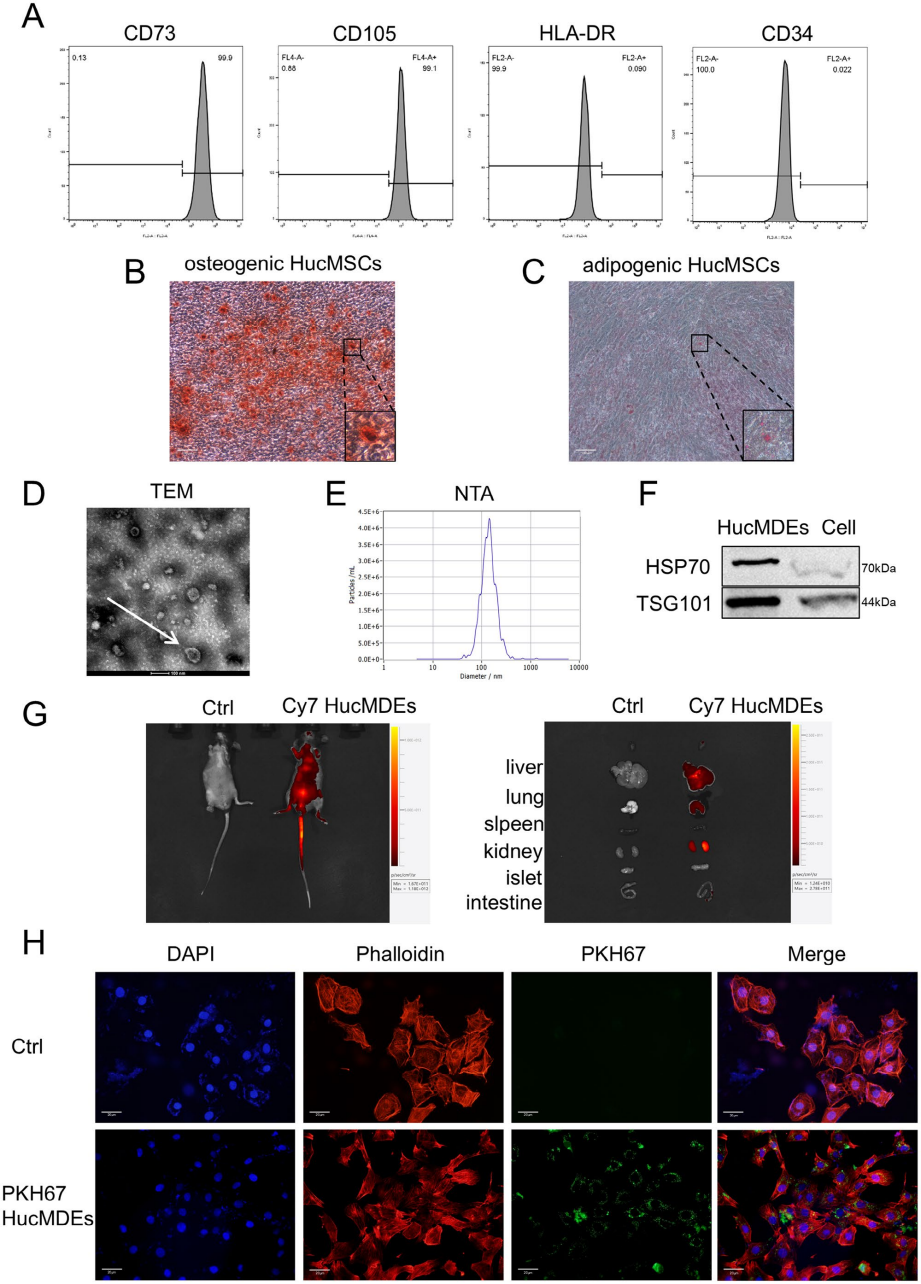
Identification of human umbilical cord HucMSCs and HucMDEs and exosome‐incorporation experiments in vivo and in vitro. (A) Stem cell surface markers as detected by flow cytometry. (B) Alizarin Red staining of osteogenic HucMSCs (scale bar, 100 μm). (C) Oil red O staining of adipogenic HucMSCs (scale bar, 100 μm). (D) The morphology of the exosomes was evaluated with TEM. (E) The size and number of exosomes were determined by NTA microscopy. (F) Detection of exosome surface markers by Western blot (WB) analysis. (G) Cy7‐labeled exosomes were imaged in mice in vivo and in organs. (H) Exosome‐incorporation experiment in AML12 cells (scale bar, 20 μm). HucMSCs, human umbilical cord mesenchymal stem cells; HucMDEs, HucMSC‐derived exosomes; TEM, transmission electron microscopy; NTA, nanoparticle tracking analyzer.

Flow cytometric analysis of HucMSC surface markers showed negligible expression of HLA‐DR and CD34 (< 1% of cells), while we observed positive expression of CD73 and CD105 (> 99% of cells), consistent with the standard immunophenotype of MSCs (Figure 1A). HucMSCs were induced to differentiate into osteoblasts. After approximately 3 weeks, we observed the formation of calcium nodules microscopically using Alizarin Red staining, and HucMSCs were induced to differentiate into osteoblasts (Figure 1B). We then conducted lipogenic induction of HucMSCs and performed Oil Red O staining. We observed a large number of lipid droplets that were colored, indicating that the HucMSCs had been induced to differentiate into adipocytes (Figure 1C).

TEM was used to visualize exosomes extracted from HucMSCs culture medium, revealing an orb‐like, vesicle‐like, double‐membrane structure (Figure 1D). Nanoparticle tracking analysis (NTA) showed that the mean diameter of the isolated vesicles was 144 nm, within the typical size range of exosomes (Figure 1E). The extracted exosomes were phenotypically verified by western blot (WB) analysis, revealing that the exosome surface markers HSP70 and TSG101 were highly expressed on the exosomes and expressed at lower levels in the cells (Figure 1F). In vivo fluorescence imaging showed that Cy7‐labeled HucMDEs were predominantly enriched in the mouse liver after tail vein injection (Figure 1G). In vitro cellular uptake assays confirmed that exosomes were internalized by AML12 cells and distributed throughout the cytoplasm (Figure 1H).

### 3.2 HucMDEs Improve Glucose Tolerance, Increase Insulin Sensitivity, and Relieve Liver Dysfunction in Aging Mice

**FIGURE 2:**
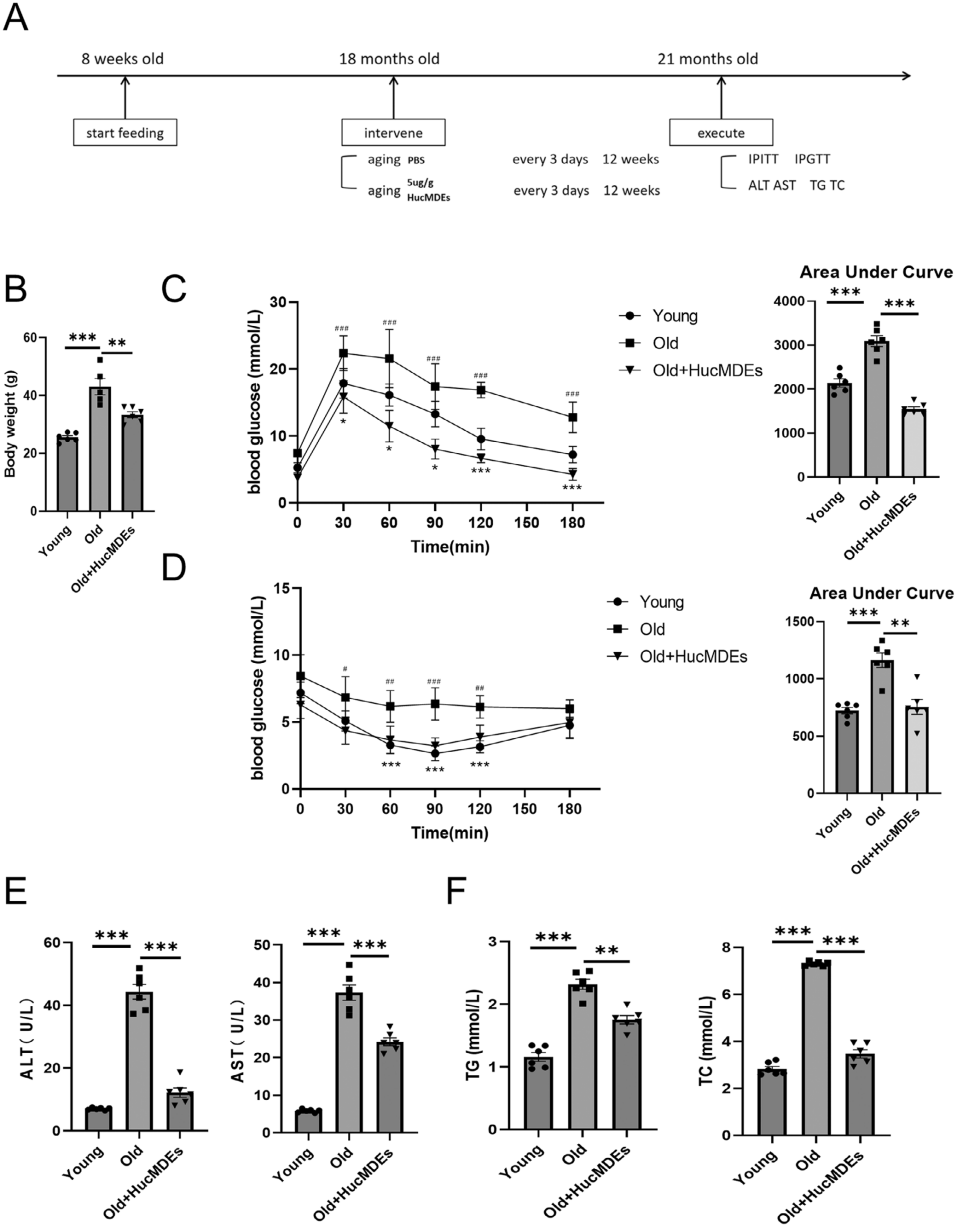
HucMDEs improved body weight, islet function, liver function, and lipid levels in aging mice. The mice were divided into three groups: a young group (Young), an old group (Old), and an old group treated with HucMDEs (Old+HucMDEs). (A) Timeline of animal experiments. (B) Weight changes in the three groups of mice. (C) IPGTT and AUC. *n* = 6 in Young, *n* = 6 in Old, *n* = 6 in Old+HucMDEs. (D) IPITT and AUC. (E) Changes in ALT and AST levels in diverse groups of mice. (F) Changes in triglyceride (TG) and total cholesterol (TC) levels in the serum of mice. *n* = 6 in Young, *n* = 6 in Old, *n* = 6 in Old+HucMDEs. Young vs. Old, *; Old versus Old+HucMDEs, #. All data are expressed as the mean ± SEM. *p < 0.05; **p < 0.01; ***p < 0.001; ^#^ *p* < 0.05; ^##^ *p* < 0.01; and ^###^ *p* < 0.001. IPGTT, Intraperitoneal glucose tolerance test; IPITT, Intraperitoneal insulin tolerance test; AUC, area under the curve; ALT, alanine aminotransferase; AST, aspartate aminotransferase.

The design of the animal experiment is shown in Figure 2A. To explore the effect of HucMDEs on body weight, we measured the body weights of mice in the Young, Old, and Old+HucMDEs groups. Significant differences in body weight were observed among the three groups: the average body weight of the mice in the Old group was higher than that of those in the Young group (*p* < 0.001, Figure 2B), while the body weights of the Old mice injected with exosomes decreased (*p* < 0.01, Figure 2B).

IPGTT results showed that the Old group peaked faster than the Young group and possessed a higher peak value than the Young group. Moreover, blood glucose concentrations fell more slowly and still did not return to normal levels after 180 min, and the area under the curve (AUC) was greater in the Old group than in the Young group (*p* < 0.001, Figure 2C). IPITT assays revealed that blood glucose in the Old group declined slowly, that there was no apparent glucose nadir within 180 min, that insulin sensitivity was lower in the Old group than in the Young group, and that the AUC was greater in the Old group than in the Young group (*p* < 0.001, Figure 2D). Compared with the mice in the Old group, the Old+HucMDEs group showed a lower and delayed glucose peak, faster glucose clearance (returning to near‐baseline levels at 120 min), and a significantly reduced IPGTT AUC (*p* < 0.001, Figure 2C). In the IPITT assay, HucMDE treatment accelerated blood glucose reduction (reaching a nadir at 90 min), improved insulin sensitivity, and significantly decreased the IPITT AUC (*p* < 0.01, Figure 2D). These results indicate that the insulin sensitivity of aging mice was decreased and that HucMDEs treatment can ameliorate insulin resistance and reduce body weight in aged animals.

To investigate the impact of exosomes on liver function and serum lipids in aging mice, we assessed the levels of alanine aminotransferase (ALT), aspartate aminotransferase (AST), triglyceride (TG), and total cholesterol (TC) in the three groups of mice (Figure 2E,F). ALT, AST, TG, and TC levels in the Old group were all significantly higher than those in the Young group (*p* < 0.001, Figure 2E), while ALT and AST levels in the Old+HucMDEs group were decreased compared with levels in the Old group. The TG and TC levels in the Old+HucMDEs group also declined compared with those in the Old group, with the reduction in TC being most pronounced (*p* < 0.001, Figure 2E).

The above results demonstrate that aging induces liver dysfunction and dyslipidemia in mice, and that HucMDE administration can effectively improve liver function and normalize serum lipid levels in aging mice.

### 3.3 HucMDEs Improve Liver Senescence and Promote Lipid Metabolism and Glycogen Synthesis in Aging Mice

**FIGURE 3:**
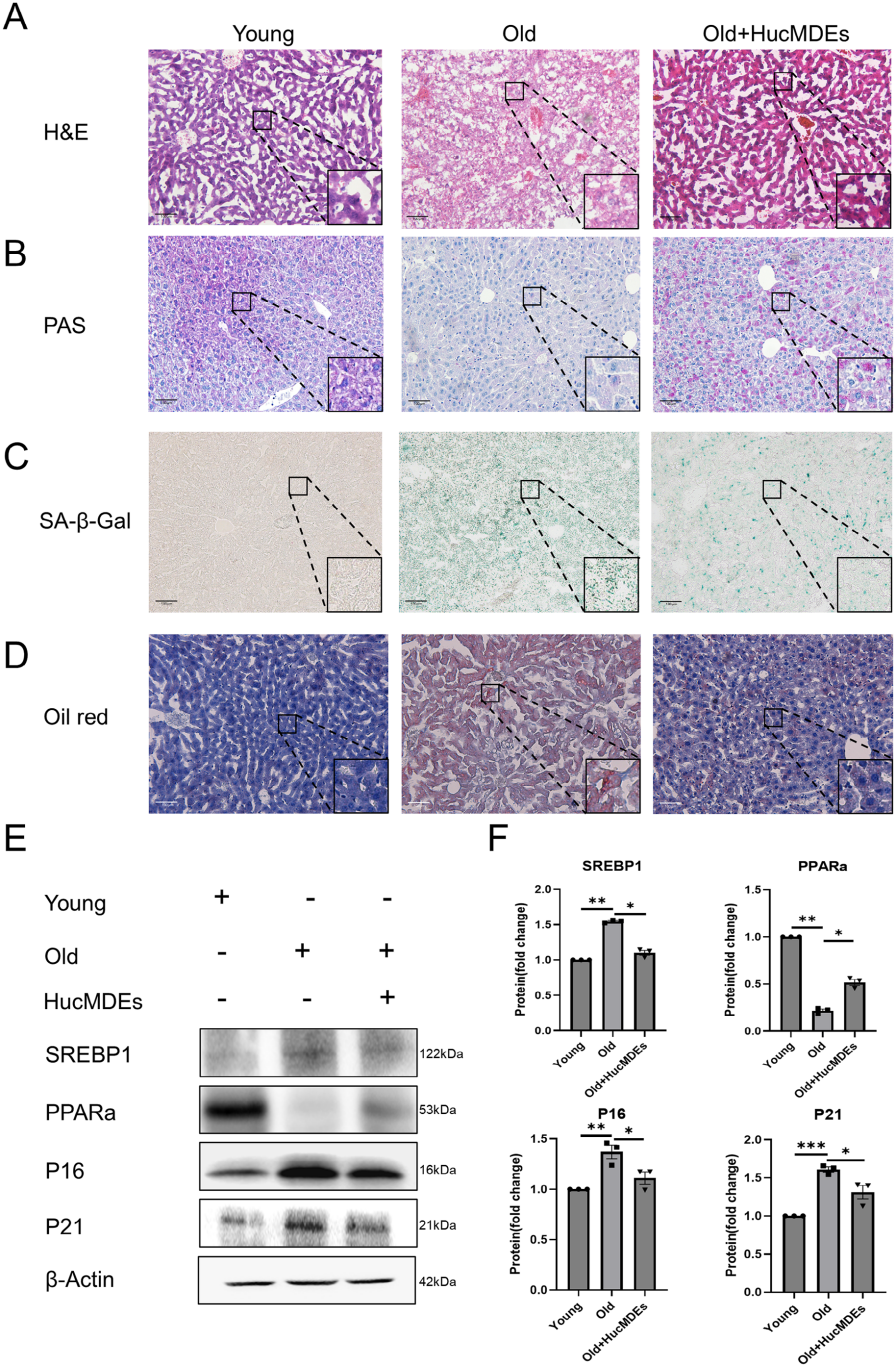
HucMDEs alleviate liver senescence and improve lipid metabolism in aging mice. (A–D) Liver H&E, SA‐β‐Gal, PAS, and oil red O staining in the different groups of mice. (E–F) The expression of key lipid metabolism‐ and senescence‐related proteins in the livers of mice was assessed by WB and statistical analyses All data are expressed as the mean ± SEM. *p < 0.05; **p < 0.01; and ***p < 0.001 (scale bar, 100 μm). H&E, hematoxylin–eosin; SA‐β‐Gal, senescence‐associated‐β‐galactosidase; PAS, periodic acid‐Schiff.

H&E staining revealed that the liver tissue structure of the Old group was more structurally disorganized than that of the Young group (Figure 3A). HucMDE treatment partially restored hepatic tissue structure, with hepatocytes arranged more orderly and exhibiting normal morphology. Periodic acid‐Schiff (PAS) staining revealed that liver glycogen synthesis was reduced in the aging group and increased in the Old+HucMDEs group (Figure 3B). Senescence‐associated‐β‐galactosidase (SA‐β‐Gal) staining showed that β‐galactosidase coloration in the liver in the Old group increased compared with that in the Young group and that the dye color of the liver in the Old+HucMDEs group was reduced compared with the aged group (Figure 3C). Oil Red O staining showed that lipid deposition was higher in the Old group than in the Young group and that liver lipid deposition was lower in the Old+HucMDEs group than in the Old group (Figure 3D). Quantitative analysis is provided in Data S1 (Figure S1).

We then examined liver lipid metabolism and the expression of age‐related proteins in each group of mice (Figure 3E,F). Compared with the Young group, in the Old group, the expression of SREBP1, a key protein in lipid synthesis, was significantly elevated (*p* < 0.01), and the expression of PPARα, a key protein in lipid catabolism, was significantly downregulated (*p* < 0.01). HucMDE treatment significantly decreased SREBP1 expression (*p* < 0.05) and increased PPARα expression (*p* < 0.05) in aged mouse livers. The expression levels of the senescence‐associated secretory phenotypes (SASPs) markers P16 and P21 were higher in the Old group than in the Young group (*p* < 0.01), and the expression levels of P16 and P21 were lower in the Old+HucMDEs group than in the Old group (*p* < 0.05).

These results indicate that the structure and function of the liver in aging mice were disrupted, and that HucMDEs alleviated liver aging in mice, promoting lipid metabolism and glycogen synthesis.

### 3.4 HucMDEs Improve Lipid Metabolism and Senescence and Promote Glycogen Synthesis in PA‐Stimulated AML12 Cells

**FIGURE 4:**
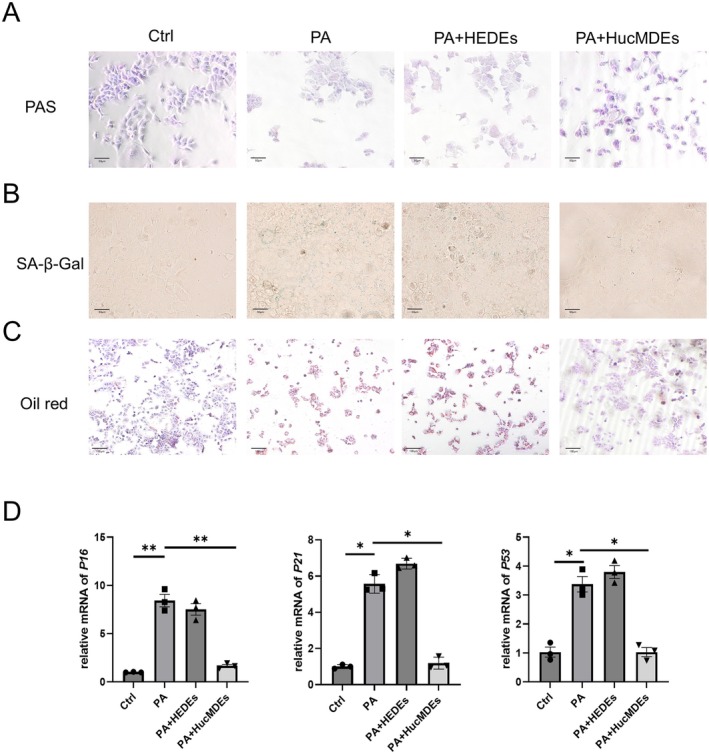
HucMDEs alleviate PA‐induced senescence in AML12 cells. (A–C) SA‐β‐Gal, PAS, and oil red O staining in the different groups of AML12 cells (scale bar, 50/100 μm). (D) The expression of key senescence genes in AML12 cells was determined by quantitative real‐time PCR (qPCR). All data are expressed as the mean ± SEM. *p < 0.05; **p < 0.01. qPCR, quantitative real‐time PCR.

**FIGURE 5:**
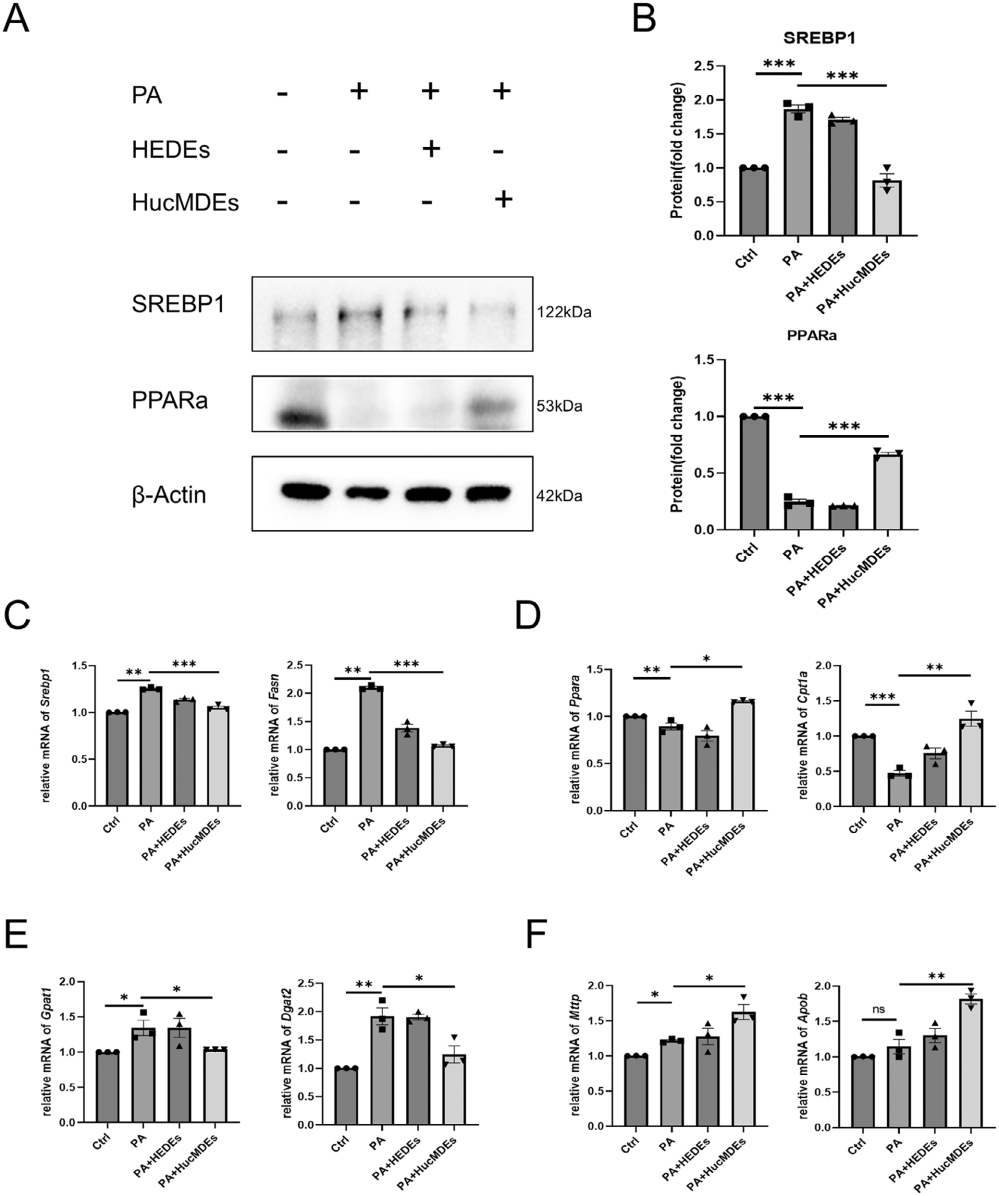
HucMDEs alleviate PA‐induced lipid metabolism in AML12 cells. (A, B) The expression of key lipid metabolism proteins in AML12 cells was determined by WB and statistical analyses. (C–F) The expression of key lipid synthesis, lipolysis, triglyceride synthesis, and lipid transport genes in AML12 cells was determined by qPCR. All data are expressed as the mean ± SEM. *p < 0.05; **p < 0.01; and ***p < 0.001.

We further investigated the effects of HucMDEs on PA‐stimulated AML12 cells in vitro. We treated AML12 cells with palmitic acid (PA), HEDEs, or HucMDEs. Our oil red O staining results showed that the staining intensity was higher in the PA and PA+HEDEs groups than in the Ctrl group and that the staining in the PA+HucMDEs group was less intense than that in the PA group or PA+HEDEs group (Figure 4C). Quantitative data are provided in Data S1 (Figure S2). To further explore the effect of HucMDEs on lipid metabolism in cells, we examined the expression of key genes involved in lipolysis, triglyceride synthesis, and lipid transport in AML12 cells under different treatments. WB revealed no difference in the expression of SREBP1, a lipid synthesis gene, or PPARα, a lipid catabolism gene, between the PA and PA+HEDEs groups (Figure 5A,B). PA treatment significantly upregulated SREBP1 expression compared with the control (*p* < 0.001), while HucMDE co‐treatment reduced SREBP1 levels to near‐control levels (*p* < 0.001 vs. PA group). Furthermore, PPARα expression was lower in the PA group than in the Ctrl group (*p* < 0.001). The expression of PPARα was higher in the PA+HucMDE group than in the PA group (*p* < 0.001), but it was still lower than that in the Ctrl group (Figure 5A,B). qPCR results revealed that mRNA levels of the lipogenic genes *Srebp1* and *Fasn* were higher in the PA group than in the Ctrl group (*p* < 0.01) and lower in the PA + HucMDEs group (*p* < 0.001, Figure 5C), while the expression of the lipolytic genes *Ppara* and *Cpt1a* was decreased in the PA group (*p* < 0.01) and increased in the PA + HucMDEs group (*p* < 0.05, Figure 5D). The expression of *Gpat1* and *Dgat2*, key genes involved in TG synthesis, was higher in the PA group than in the Ctrl group (*p* < 0.05) and lower in the PA+ HucMDEs group (*p* < 0.05, Figure 5E). Additionally, the expression levels of the lipid transport genes *Mttp* and *Apob* were higher in the PA+ HucMDEs group than in the PA group (*p* < 0.05, Figure 5F).

SA‐β‐Gal staining results showed that PA treatment induced significant cellular senescence, as evidenced by increased staining intensity, while HucMDE co‐treatment markedly reduced SA‐β‐Gal positivity (Figure 4B). To further explore the effect of HucMDEs on cellular senescence, we evaluated the mRNA expression of key SASP genes in AML12 cells in different treatment groups (Figure 4D). The expression levels of *P16*, *P21*, and *P53* were significantly (*p* < 0.05) higher in the PA groups than in the Ctrl group. The expression levels of *P16*, *P21*, and *P53* were significantly (*p* < 0.05) lower in the PA+HucMDEs group than in the PA group (Figure 4D). PAS staining revealed that intracellular glycogen content was reduced in PA‐treated cells compared with controls, and HucMDE co‐treatment restored glycogen levels (Figure 4A).

Collectively, these results demonstrate that HucMDEs can reduce lipogenesis, promote lipolysis and lipid transport, suppress triglyceride synthesis, alleviate cellular senescence, and restore glycogen synthesis in PA‐stimulated hepatocytes.

### 3.5 HucMDEs Increase Liver Autophagy Levels In Vivo and In Vitro

**FIGURE 6:**
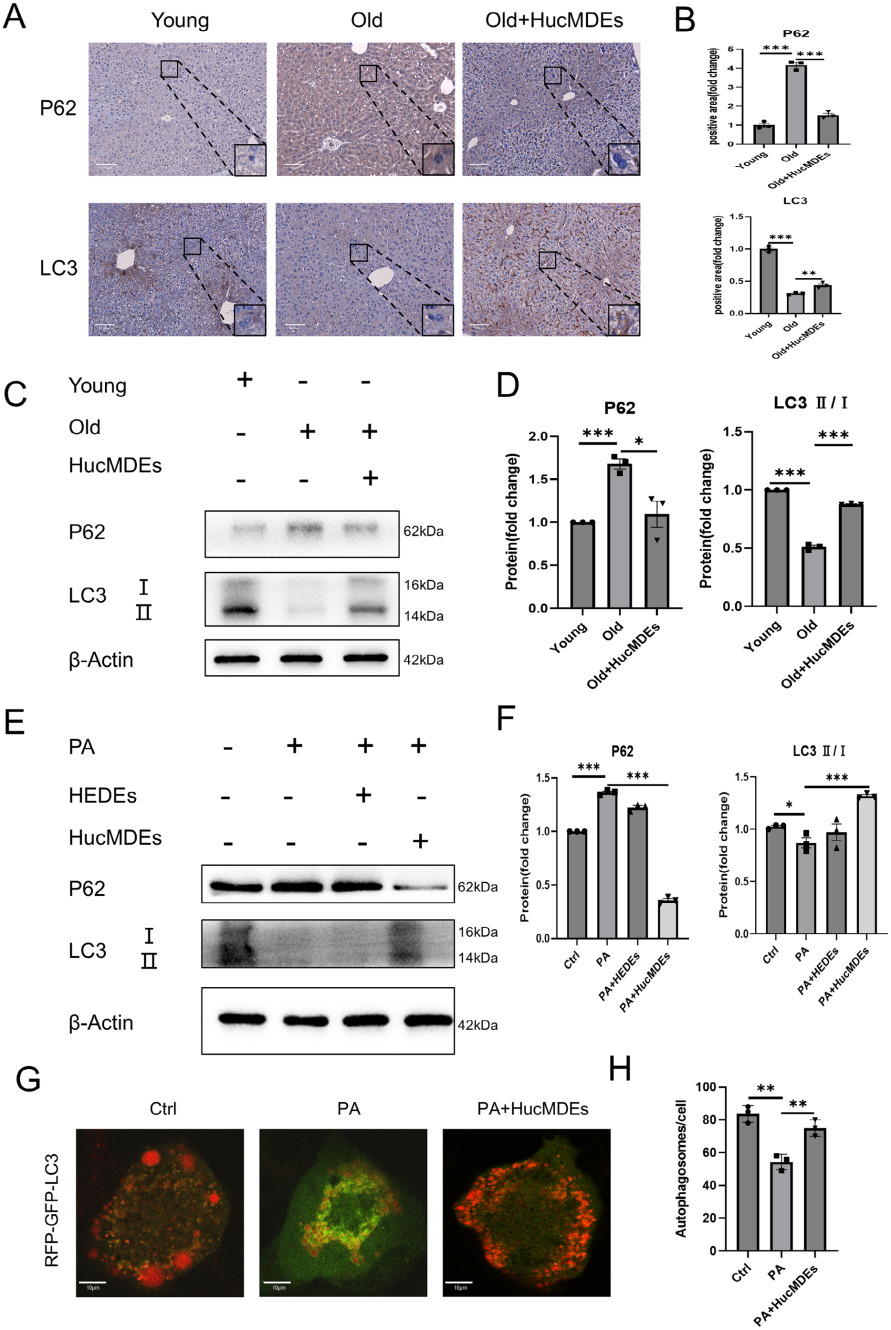
HucMDEs enhance autophagy in liver and AML12 cells. (A–D) The expression levels of key autophagic proteins in the mouse liver were detected by IHC, WB, and statistical analyses. (E–F) The expression levels of key autophagic proteins in AML12 cells were assessed by WB and statistical analyses. (G–H) Immunofluorescence images showing autophagy flux labeled by RFP‐GFP‐LC3 in AML12 cells after PA and HucMDEs treatment (scale bar, 10 μm). All data are expressed as the mean ± SEM. *p < 0.05; **p < 0.01; and ***p < 0.001.

To investigate the mechanism by which HucMDEs exert the effects mentioned above, we assessed autophagy‐related gene and protein expression both in vivo and in vitro. In vivo, we detected the expression levels of P62 and LC3 proteins in liver tissues from different groups by immunohistochemical staining (IHC) and WB (Figure 6A–D). The immunohistochemical results showed that the level of LC3 decreased in the Old group and increased in the Old + HucMDEs group, whereas that of P62 showed the opposite trend (Figure 6A,B). The WB results showed that the expression of the autophagy‐related protein P62 in the Old group was increased compared with the Young group (*p* < 0.001, Figure 6C,D). Furthermore, P62 expression decreased in the Old+HucMDEs group compared with that in the Old group (*p* < 0.05, Figure 6C,D). The LC3 II/I ratio was significantly reduced in the Old group compared with the Young group. HucMDE treatment significantly increased the LC3‐II/I ratio in aged mouse livers, though it did not fully restore to young control levels (*p* < 0.001, Figure 6C,D).

In vitro, compared with that in the control group, P62 protein expression was increased in PA‐stimulated AML12 cells (*p* < 0.001, Figure 6E,F). Additionally, compared with the PA group, the protein expression of P62 in the PA+HucMDEs group was significantly decreased (*p* < 0.001, Figure 6E,F). Similarly, the LC3‐II/I ratio was decreased in the PA group (*p* < 0.05) and significantly elevated by HucMDE co‐treatment (*p* < 0.001, Figure 6E,F).

To further confirm the effect of HucMDEs on autophagy dynamics, we evaluated the autophagic flux both in the presence and absence of HucMDEs by RFP‐GFP‐LC3 in AML12 cells (Figure 6G). The results showed that, compared with that in the Ctrl group, the number of autophagosomes in the HucMDEs group was higher than that in the PA group (*p* < 0.01, Figure 6H).

Taken together, these results demonstrate that autophagy is impaired in the liver of aged mice and in PA‐stimulated AML12 cells, and that HucMDEs can reverse this impairment and enhance hepatocellular autophagy.

### 3.6 HucMDEs Reverse the Reduction in Autophagy in AML12 Cells

**FIGURE 7:**
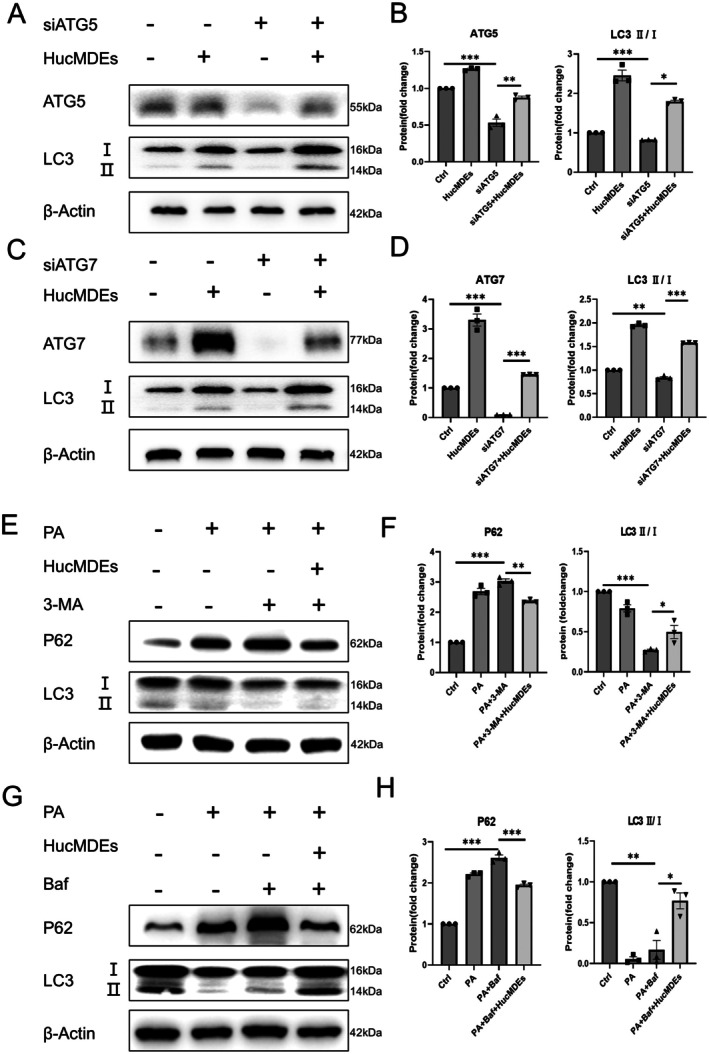
HucMDEs reverse the diminution in autophagy in AML12 cells. (A–D) Changes in autophagy levels in AML12 cells with or without knockdown of ATG5 and ATG7 were determined by WB and statistical analyses. (E–H) The expression levels of key autophagic proteins in AML12 cells treated or untreated with 3‐MA and Baf were determined by WB and statistical analyses. All data are expressed as the mean ± SEM. *p < 0.05; **p < 0.01; and ***p < 0.001. ATG5, autophagy‐related gene 5; ATG7, autophagy‐related gene 7; 3‐MA, 3‐methyladenine; Baf, bafilomycin.

To further address whether autophagy was required for the effects of HucMDEs on hepatocytes, we used siRNA‐targeted knockdown of autophagy‐related genes and determined changes in the expression of key autophagy proteins in AML12 cells by WB (Figure 7A–D).

As shown below, the expression level of ATG5 in AML12 cells after transfection with siRNA was significantly attenuated in the siATG5 group compared with that in the Ctrl group (*p* < 0.001, Figure 7A,B), and that in the HucMDEs intervention group was higher than that in the siATG5 group (*p* < 0.01, Figure 7A,B). The LC3‐II/I ratio showed a similar expression pattern across groups (Figure 7A,B). Moreover, ATG7 expression in AML12 cells after the siRNA transfection was significantly decreased (*p* < 0.001, Figure 7C,D), and HucMDE treatment significantly upregulated ATG7 levels in siATG7 cells (*p* < 0.001, Figure 7C,D). The LC3‐II/I ratio followed the same trend as ATG7 across groups (Figure 7C,D).

We further used the autophagy initiation inhibitor 3‐methyladenine (3‐MA) and the lysosomal acidification inhibitor bafilomycin A1 (Baf) to validate the regulatory effect of HucMDEs on autophagic flux (Figure 7E–H). As shown, compared with the PA + 3‐MA group, the expression level of P62 was significantly decreased in both the Ctrl group and the HucMDEs intervention group (*p* < 0.01, Figure 7E,F). The LC3‐II/I ratio was lower in the PA+3‐MA group than in the control group, while HucMDE treatment significantly increased the LC3‐II/I ratio in the presence of 3‐MA (*p* < 0.05, Figure 7E,F). Similarly, p62 levels were markedly lower in the control and HucMDE‐treated groups than in the PA+Baf group (*p* < 0.001, Figure 7G,H). The LC3‐II/I ratio was reduced in the PA+Baf group compared with controls, and HucMDE treatment significantly increased the LC3‐II/I ratio in Baf‐treated cells (*p* < 0.05, Figure 7G,H).

The above results indicate that HucMDEs can rescue autophagy impairment induced by ATG gene knockdown, autophagy initiation inhibition, or lysosomal dysfunction in hepatocytes, further confirming that HucMDEs enhance hepatocellular autophagic flux.

### 3.7 THBS1 Is Identified as a Critical Effector Protein in HucMDEs

**FIGURE 8:**
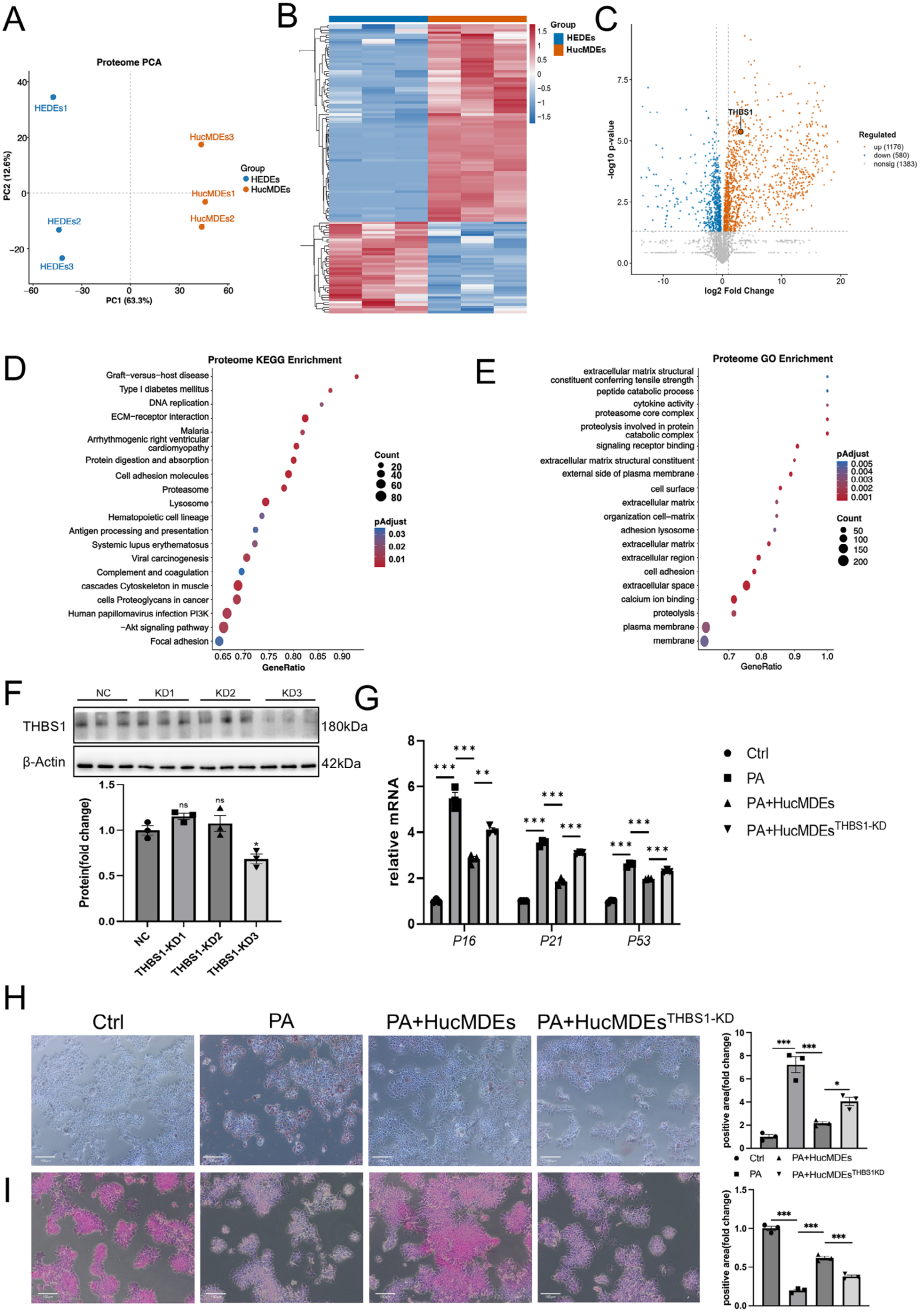
Proteomic identification of THBS1 as a key effector protein in HucMDEs. (A–B) PCA and heatmap of comparative proteomic analysis between HucMDEs and HEDEs. (C) Volcano plot showing differentially expressed proteins between HucMDEs and HEDEs. GO (D) and KEGG (E) enrichment analysis of upregulated proteins. (F) Validation of THBS1 knockdown efficiency in HucMSCs by western blotting. Effects of THBS1‐depleted HucMDEs on cellular senescence (G), Oil Red O staining (H), and PAS staining (I) in PA‐stimulated AML12 cells. Scale bar, 100 μm. Data are expressed as mean ± SEM. *p < 0.05; **p < 0.01; and ***p < 0.001. THBS1, thrombospondin‐1; GO, Gene Ontology; KEGG, Kyoto Encyclopedia of Genes and Genomes; HEDEs, HELF‐derived exosomes.

To identify the functional cargo responsible for the beneficial effects of HucMDEs on aging‐related hepatic lipid metabolism, we performed comparative proteomic analysis between HucMDEs and human embryonic lung fibroblast (HELF)‐derived exosomes (HEDEs) (Figure 8A,B). The volcano plot revealed a distinct protein expression profile, with thrombospondin‐1 (THBS1) identified as one of the most significantly enriched proteins in HucMDEs (Figure 8C). Gene ontology (GO) and Kyoto Encyclopedia of Genes and Genomes (KEGG) enrichment analyses demonstrated that the upregulated proteins were predominantly associated with “extracellular matrix organization”, “cell‐matrix adhesion”, “lysosome”, “ECM‐receptor interaction”, “adhesion sites”, and “PI3K‐Akt signaling pathway” (Figure 8D,E). Notably, the concurrent enrichment of lysosomal and proteasomal pathways is consistent with our functional data showing that HucMDEs enhance autophagic flux, while the PI3K‐Akt pathway is a well‐established regulator of both autophagy and cellular senescence. To validate the functional importance of THBS1, we performed shRNA‐mediated knockdown of THBS1 in HucMSCs (Figure 8F) and isolated the corresponding exosomes (HucMDEs^THBS1‐KD^). Strikingly, THBS1‐depleted HucMDEs failed to reduce lipid deposition, restore glycogen synthesis, and alleviate cellular senescence in PA‐stimulated AML12 cells (Figure 8G–I), establishing THBS1 as a critical effector protein within HucMDEs.

### 3.8 PPAR Signaling Is Identified as a Downstream Mediator of HucMDEs

**FIGURE 9:**
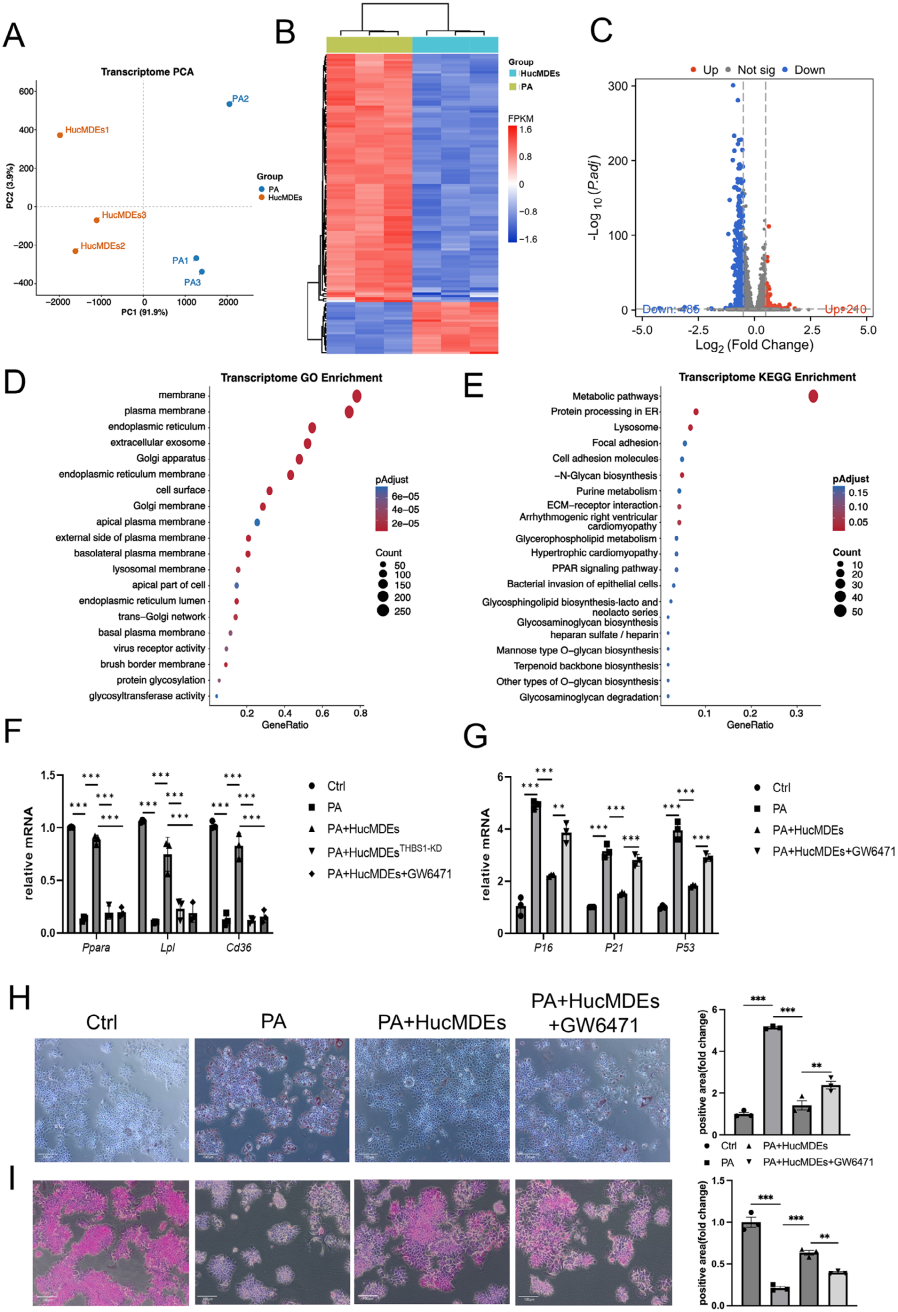
RNA‐seq identifies PPAR as a downstream signaling pathway mediating the effects of HucMDEs. (A, B) PCA and heatmap of RNA‐seq analysis in PA‐treated AML12 cells with or without HucMDEs intervention. (C) Volcano plot of differentially expressed genes (DEGs) between PA and PA+HucMDEs groups. GO (D) and KEGG (E) enrichment analysis of DEGs. (F) Relative mRNA expression of PPARα pathway‐related genes in AML12 cells treated with PA, PA+HucMDEs, PA+HucMDEs+GW6471, or PA+HucMDEs^THBS1‐KD^. Effects of GW6471 on cellular senescence (G), Oil Red O staining (H), and PAS staining (I) in PA‐stimulated AML12 cells with or without HucMDEs intervention. Scale bar, 100 μm. Data are expressed as mean ± SEM. **p < 0.01; ***p < 0.001. DEGs, differentially expressed genes.

To further elucidate the downstream signaling pathway through which THBS1‐enriched HucMDEs improve hepatic metabolism, we performed RNA‐seq on PA‐treated AML12 cells with or without HucMDEs intervention (Figure 9A,B). A total of 695 differentially expressed genes (DEGs) were identified, of which 210 were up‐regulated and 485 were down‐regulated (Figure 9C). GO enrichment analysis revealed that the DEGs were significantly enriched in categories including “extracellular exosome”, “cell surface”, and “lysosomal membrane”, etc. (Figure 9D). KEGG pathway enrichment analysis identified the PPAR signaling pathway as one of the most significantly enriched pathways (Figure 9E). To determine whether PPAR is required for the effects of HucMDEs, we treated PA‐stimulated AML12 cells with the selective PPARα antagonist GW6471 (S2798, Selleck, Houston, USA). The results showed that both GW6471 and THBS1 knockdown significantly suppressed the expression of PPARα pathway‐related genes (Figure 9F). Pharmacological inhibition of PPARα markedly attenuated the protective effects of HucMDEs on lipid metabolism, glycogen synthesis, and cellular senescence, as evidenced by increased lipid deposition, reduced glycogen storage, elevated senescence marker levels (Figure 9G–I). Collectively, these results establish PPAR as an essential downstream mediator of HucMDEs‐induced amelioration of aging‐related hepatic lipid metabolism.

## 4 Discussion

No specific pharmacotherapy is currently approved for NAFLD, making the identification of effective therapeutic targets a critical research priority (Xu et al. 2022). In this study, we established a natural aging mouse model in vivo and a PA‐induced cellular model in vitro to investigate the therapeutic effects of exosomes derived from HucMSCs on aging‐related hepatic lipid dysregulation and its underlying mechanisms. We found that HucMDE administration reduced hepatic lipid deposition, improved lipid metabolism, and elevated autophagy levels. Consistent in vitro results showed that HucMDEs ameliorated lipid metabolism disorders and restored autophagy in PA‐stimulated hepatocytes.

The first stage of NAFLD is characterized by the accumulation of fat in hepatocytes. It has been suggested that the increase in the prevalence of NAFLD in the aging human population indicates a link between cellular senescence and lipid deposition (Hardy et al. 2016). One experiment in rats with NAFLD showed that the greater the accumulation of subcutaneous fat, the greater the expression of the senescence‐related markers P16 and P21 (Xiyuan Zhang et al. 2012); our in vitro and in vivo results were consistent with this finding. Mice in the aging state are more likely to accumulate fat in liver cells and express genes related to mitochondrial dysfunction, including PPARα, the key gene in lipolysis (Wan et al. 2020). In the present study, the expression of PPARα was downregulated in the aging group of mice, consistent with the results of previous studies. It has been suggested that metabolic disorders also accelerate cellular senescence in metabolism‐related tissues and organs (Papatheodoridi et al. 2020). During the progression of NAFLD, adipocytes oversecrete inflammatory factors such as TNFα and IL‐6 in concert with increased free fatty acids to aggravate inflammatory responses and oxidative stress, leading to liver injury. Moreover, free fatty acids can directly induce cellular senescence and exacerbate lipid deposition (Qin et al. 2020). Some studies have indicated that senescent cells secrete SASPs, which include the proinflammatory factors IL‐6 and IL‐8 (Schafer et al. 2016). These findings suggest that cellular senescence and inflammation are tightly interconnected and both contribute critically to NAFLD progression.

MSC‐derived exosomes play a crucial role in delaying aging and treating age‐related disorders, including cardiovascular disease, neurodegeneration, skin aging, testicular ovarian functions, and sarcopenia. MSCs‐derived exosomes can treat age‐related diseases through mechanisms, including modulation of oxidative stress and regulation of the mTOR, P53, and SIRT1 signaling pathways (Li and Bai 2025; Li, Huang, et al. 2023; Zhang et al. 2023). Recent studies on the role of MSC‐derived exosomes in age‐related liver diseases are currently primarily focused on the treatment of liver fibrosis and hepatocellular carcinoma (Gao et al. 2023). There are relatively few studies on the regulation of hepatic metabolism by exosomes in the aging status (Lou et al. 2017), whether MSC‐exosomes exert similar effects in the context of natural aging—a condition characterized by progressive autophagy decline and exacerbated lipid dysregulation—remains largely unexplored. In the present study, we first demonstrate that HucMDEs improve aging‐related hepatic lipid metabolism disorders by enhancing autophagy.

A variety of mechanisms, including endoplasmic reticulum stress, mitochondrial dysfunction, hypoxia, and cell death, influence the regulation of hepatic lipid metabolism in NAFLD (Geng et al. 2021). It has been noted that the efficiency of autophagy declines with age (Kitada and Koya 2021). Autophagy—particularly selective autophagy in dysfunctional mitochondria—has been suggested to constitute a protective mechanism in NAFLD; furthermore, it can help restore hepatocyte numbers to prevent NAFLD (Wang et al. 2015). Researchers have also demonstrated that hepatocyte senescence in mice with NAFLD can be mitigated by restoring autophagy (Huang et al. 2024; Zhang et al. 2022). GDF11 may inhibit the intensification of autophagy activity through the mTORC1/TFEB signaling pathway, thereby accelerating liver aging (Sun et al. 2022). The findings of the present study corroborated this report, with our results showing that elevated hepatic autophagy declined in aging mice and that hepatic lipid metabolism was improved after exosomal intervention. Previous studies have demonstrated that lipid accumulation exacerbates liver aging, which is ameliorated by activating AMPK/ULK1 and inhibiting Akt/mTOR/ULK1 pathways. These findings are consistent with the phenomena observed in this study, and they suggest a possible mechanism by which HucMDEs reduce liver lipid metabolism under aging conditions by altering autophagy levels (Gao, Zhang, et al. 2020; Li et al. 2025). Some studies have also shown that autophagy levels are higher in younger livers than in aged livers, and that enhanced autophagy promotes liver cell regeneration (Liu et al. 2018; Xu et al. 2020). Other studies have revealed that TXNIP/VDUP1 attenuates steatitis through autophagy and fatty acid oxidative catabolism (Park et al. 2021), a phenomenon also demonstrated in our in vitro experiments using PA‐stimulated liver cells. Autophagy removes lipid droplets from hepatocytes and can inhibit the production of inflammatory molecules, factors that are critical to improving hepatic lipid metabolism by supplying energy to activated hepatic stellate cells during liver fibrosis (Chen and Lin 2022). Many autophagy‐related genes are involved in the regulation of hepatic lipid metabolism, of which LC3, P62, ATG5, and ATG7 play crucial roles (Li, Lin, et al. 2023; Sakane et al. 2021). The deletion of Atg7 has been found to induce senescence‐associated β‐galactosidase activities and the SASP (Huda et al. 2022). Moreover, one study found that Atg5 KO mice exhibit enhanced hepatic accumulation of p62, and also that the expression level of p21 is associated with hepatocyte senescence and senescence‐associated β‐galactosidase expression (Toshima et al. 2014). Our data confirmed these findings. Other studies have revealed that mesenchymal stem cell‐derived exosomes ameliorate aging by activating autophagy and inhibiting ROS/NLRP3 inflammasomes via the AMPK/mTOR signaling pathway, which is consistent with our research results and suggests a potential mechanism of action for HucMDEs (Zhou et al. 2024). Studies have also demonstrated that knocking down ROC1 leads to an increase in autophagy levels in liver cells and a worsening of liver cell aging (Yang et al. 2013), this further indicating that autophagy is closely related to liver aging. However, some researchers have proposed that autophagy may play opposing roles in different cells and at different stages of liver disease; for example, autophagy plays a negative role in hepatocellular carcinoma (Allaire et al. 2019). Therefore, a regulatory role for autophagy as an underlying mechanism of action in the progression of NAFLD has not yet been fully clarified, and additional studies are needed.

To systematically identify the cargo responsible for the beneficial effects of HucMDEs, we conducted a comparative proteomics analysis and initially screened THBS1 as a candidate protein. Several studies have demonstrated that THBS1 plays a crucial role in regulating hepatic lipid metabolism and energy homeostasis. Bai et al. reported that recombinant THBS1 protein ameliorates high‐fat diet‐induced hepatic steatosis in mice by inhibiting de novo lipogenesis, an effect mediated through the CD36 receptor (Bai et al. 2020). Notably, THBS1 is a secreted protein that can be transported to target cells via exosomes. Cai et al. found that THBS1 is enriched in extracellular vesicles derived from young stem cells and can be delivered to senescent cells to restore autophagy and alleviate cellular senescence (Cai et al. 2024). These findings strongly support our discovery that THBS1 is a key effector molecule mediating the metabolic and anti‐aging effects of HucMDEs. PPARs is a master transcriptional regulator of lipid catabolism and has been increasingly recognized as a critical modulator of autophagy. Indeed, PPARs not only promotes fatty acid oxidation but also transcriptionally upregulates key autophagy‐lysosomal genes involved in lipophagy (Iershov et al. 2019). Conversely, autophagy deficiency leads to accumulation of PPAR repressors such as HDAC3 and NCoR1, resulting in impaired PPAR transcriptional activity and reduced lipid catabolism (Iershov et al. 2019). Regarding the relationship between THBS1 and PPARα, we propose that THBS1 delivered by HucMDEs may act through CD36 to initiate signaling cascades that converge on PPAR activation, or that THBS1 and PPARα operate in parallel pathways that collectively create a metabolic environment favorable for autophagy. Future studies, including co‐immunoprecipitation, luciferase reporter assays, and combined knockdown/agonist experiments, are required to directly test this hypothesis.

Collectively, our study provides several novel contributions: (1) it is the first to demonstrate that HucMDEs improve aging‐related hepatic lipid dysregulation by enhancing autophagy in a natural aging model; (2) it identifies THBS1 as a key exosomal effector protein responsible for this effect; (3) it establishes PPAR signaling as a critical downstream signaling pathway through which HucMDEs enhance autophagy and lipid catabolism; and (4) it proposes a potential crosstalk between THBS1 and PPAR in the context of exosome‐mediated hepatic protection. These mechanistic insights distinguish our work from previous reports that focused on young or diet‐induced models without addressing the natural aging context or identifying specific exosomal proteins and pathways.

Our research also has limitations. First, the specific hepatic cell types targeted by HucMDEs to exert autophagy‐dependent lipid metabolic regulation in aging conditions require further clarification. Second, although we have identified THBS1 as a key exosomal protein and PPAR signaling as an essential downstream pathway, the direct molecular interaction between THBS1 and PPARα requires further experimental validation. Future studies with more refined experimental designs will be conducted to address these issues.

In summary, this study provides a preliminary exploration of human umbilical cord MSC‐derived exosomes as a potential therapy for aging‐related NAFLD. We demonstrate that exosomes regulate hepatic lipid metabolism via autophagy enhancement, providing a theoretical basis for future translational research.

## Author Contributions

J.L. conducted the experiments and wrote the manuscript; M.G., J.F., D.H., R.H., and F.Z. analyzed and validated the data. M.D. and Q.H. designed the experiments and reviewed and approved the manuscript. All the authors have read and approved the final manuscript.

## Funding

This work was supported by the National Natural Science Foundation of China (Grant No. 82300892), the Natural Science Foundation of Shandong Province (Grant No. ZR2025MS1417 and Grant No. ZR2022MH182), and the China International Medical Foundation (Grant No. 2024‐N‐05‐05).

## Ethics Statement

All animal experimental protocols were approved by the Animal Ethics Committee of Qilu Hospital of Shandong University (DWLL‐2022‐090). All experiments were performed in accordance with relevant guidelines and regulations. The study complied with the ARRIVE guidelines. With the approval of the Ethics Committee at Qilu Hospital of Shandong University, we obtained fresh human umbilical cords from full‐term births by cesarean section and all experimental protocols (KYLL‐202008(KS)‐201). All methods were conducted in accordance with relevant guidelines and regulations.

## Consent

All participants provided informed consent for the use of the umbilical cord in this experimental study.

## Conflicts of Interest

The authors declare no conflicts of interest.

## Supporting information


**Data S1:** acel70642‐sup‐0001‐DataS1.docx.

**Data S2:** acel70642‐sup‐0002‐DataS2.pdf.

## Data Availability

The data supporting the outcomes of this investigation are available from the corresponding author on reasonable request. Data S1 and S2 are provided within the supporting information files.
