# Supplementary material for: Mesenchymal Stem Cell‐Derived Exosomes Improve Aging‐Related Changes in Liver Lipid Metabolism by Enhancing Autophagy
Source: Aging Cell. 2026 Jul 16;25(7):e70642. doi: 10.1111/acel.70642 (PMC13375943; doi:10.1111/acel.70642)

**Mesenchymal stem cell-derived exosomes improve aging-related changes in liver lipid metabolism by enhancing autophagy.**

Jinquan Li^1^, Mengqi Gao^1^, Jinke Feng^1^, Dini Huo^1^, Rui Hong^1^, Fuhua Zhang^1^, Qin He^1,2,3,4*^, Ming Dong^1,2,3,4*^

^1^Department of Endocrinology and Metabolism, Qilu Hospital of Shandong University, Jinan, Shandong, China

^2^Institute of Endocrine and Metabolic Diseases of Shandong University, Jinan, Shandong, China.

^3^Jinan Clinical Research Center for Endocrine and Metabolic Disease, Jinan, Shandong, China

^4^Key Laboratory of Endocrine and Metabolic Diseases, Shandong Province Medicine & Health, Jinan, Shandong, China.

Jinquan Li, Email: [ljqcdz@outlook.com](mailto:ljqcdz@outlook.com)

Mengqi Gao, Email: [13068606865@163.com](mailto:13068606865@163.com)

Jinke Feng, Email: [fjk13569668606@163.com](mailto:fjk13569668606@163.com)

Dini Huo, Email: [17539880996@163.com](mailto:17539880996@163.com)

Rui Hong, Email: [18597016138@163.com](mailto:18597016138@163.com)

Fuhua Zhang, Email: [fhuazhang@126.com](mailto:fhuazhang@126.com)

^*^ Correspondence:
Ming Dong, Email: [dr_dongming@email.sdu.edu.cn](mailto:dr.dongming@email.sdu.edu.cn)

Qin He, Email: [heqin9005@email.sdu.edu.cn](mailto:heqin9005@email.sdu.edu.cn)

**Antibody**

| Protein Name | Country | Supplier | Product Number |
| --- | --- | --- | --- |
| CD73 | USA | BioGems | No. 05811-60 |
| CD105 | USA | BioGems | No. 17111-80 |
| HLA-DR | USA | BioLegend | No. 327021 |
| CD34 | USA | BioLegend | No. 343505 |
| HSP70 | USA | CST | #4872 |
| TSG101 | USA | CST | #72312 |
| SREBP1 | USA | Affinity | AF6283 |
| PPARa | USA | Proteintech | No.66826-1-Ig |
| LC3 | USA | CST | #2775 |
| P62 | USA | CST | #5114 |
| P16 | UK | abcam | ab211542 |
| P21 | USA | Immunoway | YT3497 |
| ATG5 | USA | Proteintech | No.10181-2-AP |
| ATG7 | China | ABclonal | No.A19604 |
| THBS1 | USA | Proteintech | No.18304-1-AP |
| β-actin | China | Abways | No.AB0035 |

**Primer**

| Gene | Sequence (5’→3’) | |
| --- | --- | --- |
| Mus - P16 | F | GCTCTTCTGCTCAACTACGGT |
|  | R | CGATGTCTTGATGTCCCCGC |
| Mus - P21 | F | TTGTCGCTGTCTTGCACTCT |
|  | R | TAGAAATCTGTCAGGCTGGTCT |
| Mus - P53 | F | GGCAACTATGGCTTCCACCT |
|  | R | TTGAGGGGAGGAGAGTACGTG |
| Mus - SREBP1 | F | TGACCCGGCTATTCCGTGA |
|  | R | CTGGGCTGAGCAATACAGTTC |
| Mus - FASN | F | GGAGGTGGTGATAGCCGGTAT |
|  | R | TGGGTAATCCATAGAGCCCAG |
| Mus - PPARa | F | AGAGCCCCATCTGTCCTCTC |
|  | R | ACTGGTAGTCTGCAAAACCAAA |
| Mus - CPT1a | F | AGATCAATCGGACCCTAGACAC |
|  | R | CAGCGAGTAGCGCATAGTCA |
| Mus - DGAT2 | F | GCGCTACTTCCGAGACTACTT |
|  | R | GGGCCTTTATGCCAGGAAACT |
| Mus - GPAT1 | F | ACAGTTGGCACAATAGACGTTT |
|  | R | CCTTCCATTTCAGTGTTGCAGA |
| Mus - MTTP | F | TGTGAACTCCCCCTCCATCA |
|  | R | CATCCACCGGAGTTATCGCT |
| Mus - ApoB | F | TTCCAGATTGCTAGGCTCCCT |
|  | R | CGGGCTACTCTCTGGAAACT |
| Mus-LPL | F | TCTGTGTCTAACTGCCACTTCAA |
|  | R | GGCCCGATACAACCAGTCTACTA |
| Mus-CD36 | F | GCATGGTAGAGATGGCCTTACTT |
|  | R | GAGAGAGCACACACCACCATTTC |
| Mus - ACTIN | F | TGCTGTCCCTGTATGCCTCTG |
|  | R | TGATGTCACGCACGATTTCC |

**siRNA**

| Gene | Sequence(5’→3’) | |
| --- | --- | --- |
| Mus - ATG5 | F | GACGUUGGUAACUGACAAATT |
|  | R | UUUGUCAGUUACCAACGUCTT |
| Mus - ATG7 | F | GCUGCUACUUCUGCAAUGATT |
|  | R | UCAUUGCAGAAGUAGCAGCTT |
| Mus- GAPDH | F | CACUCAAGAUUGUCAGCAATT |
|  | R | UUGCUGACAAUCUUGAGUGAG |
| Mus - NC | F | UUCUCCGAACGUGUCACGUTT |
|  | R | ACGUGACACGUUCGGAGAATT |

**LV**

| Tag-name | Sequence |
| --- | --- |
| THBS1-RNAi-1 | GTGCTGCAGAATGTGAGGTTT |
| THBS1-RNAi-2 | GTGGTGATGGTGTGATCACAA |
| THBS1-RNAi-3 | GTGCCTGATGACAAGTTCCAA |

### The shRNA lentiviral particles targeting THBS1 were purchased from GeneChem Co., Ltd. (Shanghai, China).

### Fig. S1 Fig.S2

###
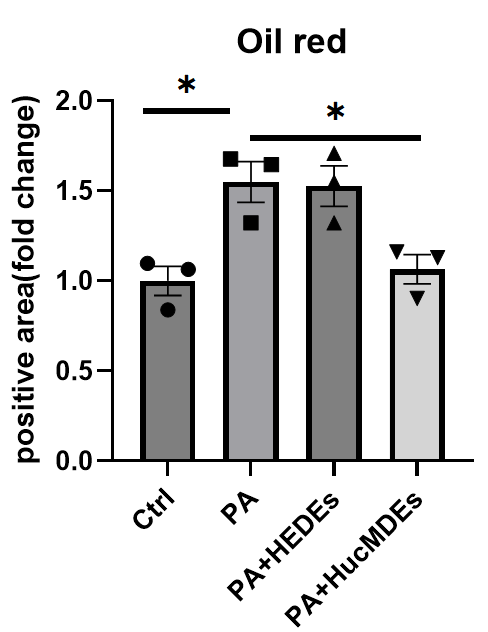

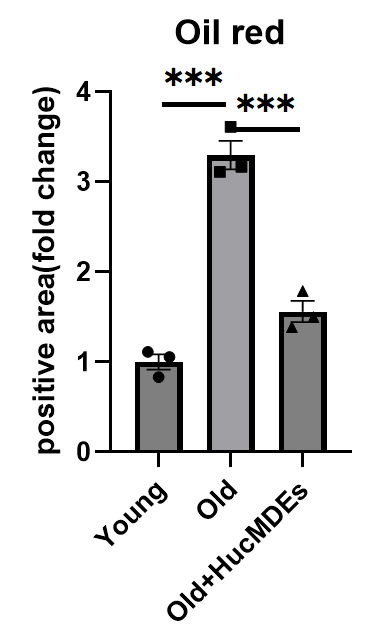

Supplement: Supplementary file 1 — Data S1: acel70642‐sup‐0001‐DataS1.docx. [file ACEL-25-e70642-s001.docx]
