# Supplementary material for: Mesenchymal Stem Cell‐Derived Exosomes Improve Aging‐Related Changes in Liver Lipid Metabolism by Enhancing Autophagy
Source: Aging Cell. 2026 Jul 16;25(7):e70642. doi: 10.1111/acel.70642 (PMC13375943; doi:10.1111/acel.70642)

**Original Western Blot**

1.Original Western Blot (WB) image for HSP70, corresponding to the data shown in Figure 1F of the manuscript.

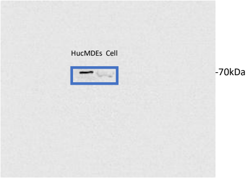


2.Original Western Blot (WB) image for TSG101, corresponding to the data shown in Figure 1F of the manuscript.

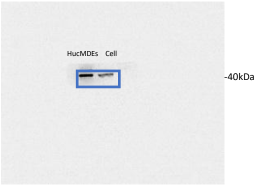


3.Original Western Blot (WB) image for SREBP1 (in blue box), corresponding to the data shown in Figure 3E of the manuscript.

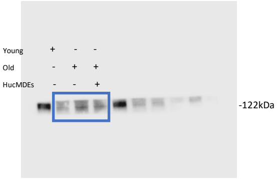


4.Original Western Blot (WB) image for PPARa (in blue box), corresponding to the data shown in Figure 3E of the manuscript.

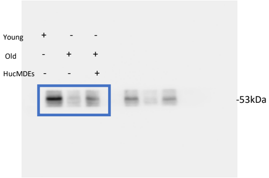


5.Original Western Blot (WB) image for P16 (in blue box), corresponding to the data shown in Figure 3E of the manuscript.

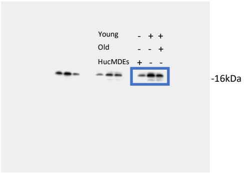


6.Original Western Blot (WB) image for P21 (in blue box), corresponding to the data shown in Figure 3E of the manuscript.

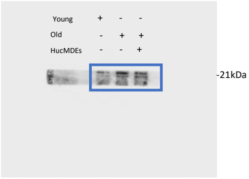

7.Original Western Blot (WB) image for β-Actin (in blue box), corresponding to the data shown in Figure 3E of the manuscript.

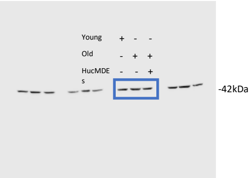


8.Original Western Blot (WB) image for SREBBP1 (in blue box), corresponding to the data shown in Figure 5A of the manuscript.

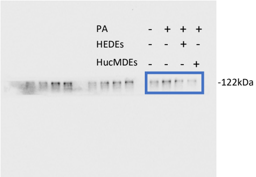


9.Original Western Blot (WB) image for PPARa (in blue box), corresponding to the data shown in Figure 5A of the manuscript.

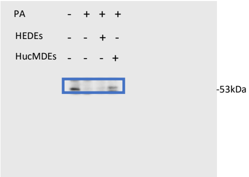


10.Original Western Blot (WB) image for β-Actin (in blue box), corresponding to the data shown in Figure 5A of the manuscript.

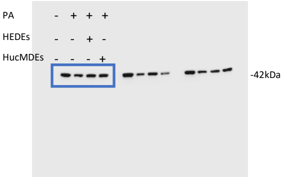


11.Original Western Blot (WB) image for P62 (in blue box), corresponding to the data shown in Figure 6C of the manuscript.

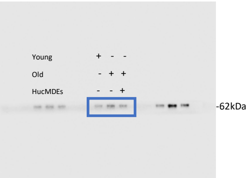


12.Original Western Blot (WB) image for LC3 (in blue box), corresponding to the data shown in Figure 6C of the manuscript.

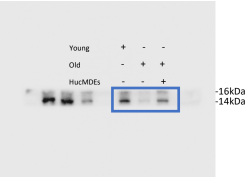

13.Original Western Blot (WB) image for β-Actin (in blue box), corresponding to the data shown in Figure 6C of the manuscript.

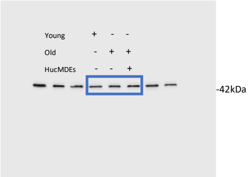


14.Original Western Blot (WB) image for P62 (in blue box), corresponding to the data shown in Figure 6E of the manuscript.

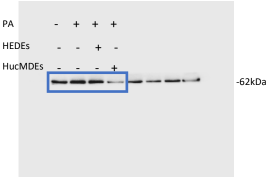


15.Original Western Blot (WB) image for LC3 (in blue box), corresponding to the data shown in Figure 6E of the manuscript.

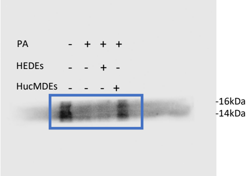


16.Original Western Blot (WB) image for β-Actin (in blue box), corresponding to the data shown in Figure 6E of the manuscript.

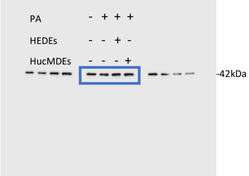


17.Original Western Blot (WB) image for ATG5 (in blue box), corresponding to the data shown in Figure 7A of the manuscript.

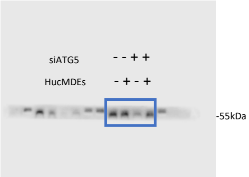


18.Original Western Blot (WB) image for LC3 (in blue box), corresponding to the data shown in Figure 7A of the manuscript.

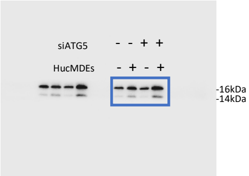

19.Original Western Blot (WB) image for β-Actin (in blue box), corresponding to the data shown in Figure 7A of the manuscript.

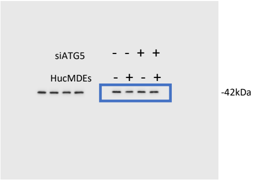


20.Original Western Blot (WB) image for ATG7 (in blue box), corresponding to the data shown in Figure 7C of the manuscript.

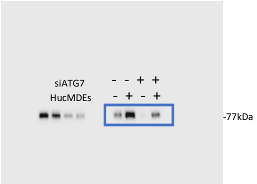


21.Original Western Blot (WB) image for LC3 (in blue box), corresponding to the data shown in Figure 7C of the manuscript.

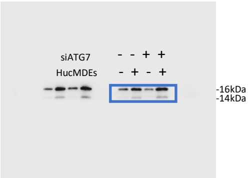


22.Original Western Blot (WB) image for β-Actin (in blue box), corresponding to the data shown in Figure 7C of the manuscript.

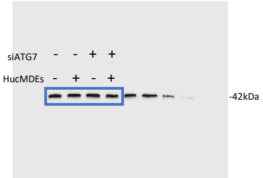


23.Original Western Blot (WB) image for P62 (in blue box), corresponding to the data shown in Figure 7E of the manuscript.

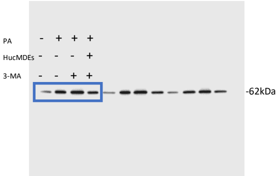


24.Original Western Blot (WB) image for  LC3 (in blue box), corresponding to the data shown in Figure 7E of the manuscript.

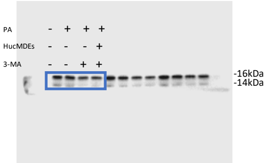

25.Original Western Blot (WB) image for β-Actin (in blue box), corresponding to the data shown in Figure 7E of the manuscript.

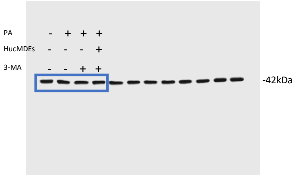


26.Original Western Blot (WB) image for P62 (in blue box), corresponding to the data shown in Figure 7G of the manuscript.

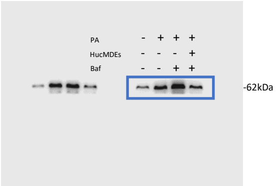


27.Original Western Blot (WB) image for LC3 (in blue box), corresponding to the data shown in Figure 7G of the manuscript.

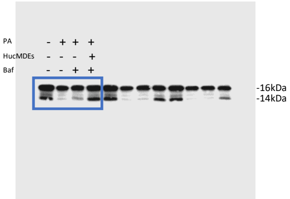


28.Original Western Blot (WB) image for β-Actin (in blue box), corresponding to the data shown in Figure 7G of the manuscript.

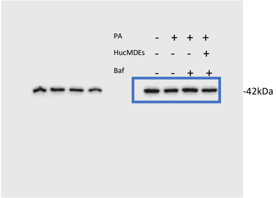


29.Original Western Blot (WB) image for THBS1 (in blue box), corresponding to the data shown in Figure 8F of the manuscript.

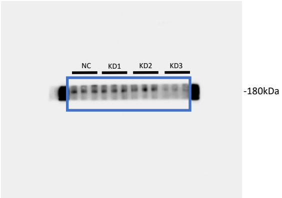


30.Original Western Blot (WB) image for β-Actin (in blue box), corresponding to the data shown in Figure 8F of the manuscript.

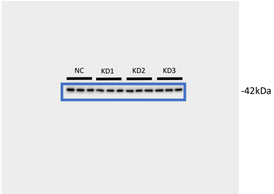

Supplement: Supplementary file 2 — Data S2: acel70642‐sup‐0002‐DataS2.pdf. [file ACEL-25-e70642-s002.pdf]
